# The frequency and clinical implication of mismatch repair protein deficiency in Chinese patients with ovarian clear cell carcinoma

**DOI:** 10.1186/s12885-022-09588-z

**Published:** 2022-04-23

**Authors:** Shuang Ye, Shuling Zhou, Siyuan Zhong, Boer Shan, Wenhua Jiang, Wentao Yang, Xu Cai, Huijuan Yang

**Affiliations:** 1 Department of Gynecologic Oncology, Fudan University Shanghai Cancer Center, Shanghai, China; 2 Department of Oncology, Shanghai Medical College, Fudan University, Shanghai, 200032 China; 3 Department of Pathology, Fudan University Shanghai Cancer Center, Shanghai, China; 4 Department of Pathology, Shanghai First Maternity and Infant Hospital, School of Medicine, Tongji University, Shanghai, China

**Keywords:** Ovarian carcinoma, Clear cell carcinoma, Mismatch repair, Clinicopathologic relevance

## Abstract

**Background:**

The aim of the present study was to assess the prevalence of deficient mismatch repair (MMR) in Chinese ovarian clear cell carcinoma (CCC) patients and its association with clinicopathologic features.

**Methods:**

Immunohistochemistry with four antibodies against MLH1, PMS2, MSH2 and MSH6 was performed on whole section slides, and the results were correlated with clinicopathologic variables.

**Results:**

A total of 108 cases were included in the present study with a median age of 52 years at first diagnosis. Early-stage disease and platinum-sensitive recurrence accounted for 62.3 and 69.6%, respectively, of the total cases. Overall, the estimated 5-year overall survival was 70.3 and 20.7% in patients with early- and late-stage tumors, respectively. Deficient MMR was identified in 5.6% (6/108) of the cohort and included MSH2/MSH6 (*n* = 4) and MLH1/PMS2 (*n* = 2). The average age of the six patients with deficient MMR was 45.6 years, and the rate of MMR-deficient tumors in women ≤50 years was relatively higher than that in women over 50 years (10.0% vs. 2.9%; *P* = 0.266). Half of the patients with deficient MMR were diagnosed with synchronous (endometrial or colorectal) and metachronous (endometrial) cancer, which was significantly more than their intact counterparts (*P* = 0.002). All six patients with deficient MMR had early-stage tumors, and the majority (83.3%) were platinum sensitive. The median progression-free survival was slightly higher in patients with defective MMR expression than in their intact counterparts (30 months vs. 27 months), but significance was not achieved (*P* = 0.471).

**Conclusions:**

Young ovarian CCC patients with concurrent diagnosis of endometrial and colorectal cancer are more likely to have MMR-deficient tumors, thereby warranting additional studies to determine whether patients harboring MMR abnormalities have a favorable prognosis.

**Supplementary Information:**

The online version contains supplementary material available at 10.1186/s12885-022-09588-z.

## Background

The histological subtypes of ovarian cancer are distinct diseases, each with different clinical and molecular characteristics [1]. Ovarian clear cell carcinoma (CCC) has unique epidemiological correlations with ethnicity, endometriosis, genetic/epigenetic alterations and specific immune-related molecular profiles [2]. In addition, ovarian CCC is challenging to treat due to its aggressiveness and chemoresistance [3]. The objective response rate of ovarian CCC to conventional chemotherapy is 9% in platinum-sensitive patients and 1% in platinum-resistant recurrence [4]. Recent clinical trials have shown that ovarian CCC patients show surprising sensitivity to immune checkpoint inhibitors, but ovarian cancer patients with high-grade serous carcinoma show modest responses [5, 6]. However, given the rarity of the disease, only small numbers of cases were included in the trials, and further verification is needed.

Mismatch repair (MMR) deficiency has been demonstrated to be a biomarker of sensitivity to immune checkpoint blockade with antibodies against programmed death receptor-1 across various types of tumors [7]. Defective MMR leads to the accumulation of mutations in the genome and microsatellite instability in tumors [8]. Lynch syndrome is characterized by loss of expression of MMR genes [9], and the most clinically significant genes are MLH1, MSH2, MSH6 and PMS2 [8, 10]. Women with Lynch syndrome are at increased risk of ovarian carcinoma, mostly clear cell and endometrioid histology [9]. In the past 5 years, several publications have focused on MMR deficiency in ovarian CCC [11–18]. The frequency of deficient MMR varies from study to study, and most studies include a small number of clear cell carcinoma patients [13–16, 18].

In the present study, we assessed the status of MMR proteins in a well-annotated unselected cohort of Chinese ovarian CCC patients. The frequency of MMR deficiency was evaluated by immunohistochemistry, and the association of MMR deficiency with clinicopathologic variables was also evaluated.

## Materials and methods

### Study population

All methods were carried out in accordance with relevant guidelines and regulations. After obtaining approval from the Institutional Review Board (050432–4-1212B), we identified all the patients by searching the surgical pathology archives for “ovarian clear cell carcinoma” from 2008 to 2018. In our institution, one surgical specimen is generally reviewed by two pathologists (one young and one senior doctor) as a routine. A third experienced pathologist will review the slides in some difficult cases or resolve discrepancies. In the present study, we included all patients with archived tissue blocks, and all available hematoxylin & eosin-stained slides were reviewed to confirm the diagnosis. Cases were excluded if they were focal carcinomas or if the clear cell component was less than 50% in mixed tumors. The requirement for written informed consent was waived considering the retrospective design of the study.

Clinicopathological information and survival outcomes were obtained from medical records. The following data were collected: the age at diagnosis of ovarian CCC; personal and family history of cancer; date and type of primary surgery; International Federation of Gynecology and Obstetrics (FIGO) stage at initial diagnosis [19]; consistent endometriosis (presence of endometriosis in the same specimen [20]); residual disease; platinum-free interval (the time interval from completion of the last platinum-based chemotherapy to disease recurrence); time of disease progression or recurrence; and tumor status at last contact. Patients were considered platinum sensitive if the platinum-free interval was more than 6 months. Progression-free survival (PFS) and overall survival (OS) were defined as the time interval from the date of the primary surgery to the date of first recurrence and death or last contact, respectively. Due to the retrospective design, some clinicopathological information was missing.

### Immunohistochemistry

Four-micrometer-thick, formalin-fixed, paraffin-embedded whole-block sections were used for immunohistochemistry. All staining was performed using an automated slide stainer (*Ventana BenchMark ULTRA*) according to our protocols as MMR protein immunohistochemistry is routinely conducted at our institution [21, 22]. The primary antibodies included anti-MLH1 (Clone G168–728), anti-MSH2 (Clone G219–1129), anti-MSH6 (Clone 44) and anti-PMS2 (Clone EPR3947) (Roche, Basel, Switzerland).

All the slides were reviewed independently by two pathologists who were blinded to the clinical information. MMR stains were interpreted as abnormal (loss of nuclear staining in all tumor cells) and normal (retained nuclear staining) [21, 22]. Lymphocytes and stromal cells served as positive internal controls.

### Statistical analyses

Continuous data are presented as the median/mean (range), and categorical data are presented as proportions. Parametric Student’s *t test*s were utilized to evaluate continuous variables, while chi-square tests (continuity correction chi-square) were used to evaluate categorical variables. Survival time was evaluated using the Kaplan–Meier model. All reported *P* values were two-sided, and *P* < 0.05 was considered statistically significant. Statistical Package for Social Science (SPSS) (Version 17.0, SPSS, Inc., Chicago, IL, USA) was used for the statistical analyses, and GraphPad Prism (Version 5.0, GraphPad Software, Inc., La Jolla, CA, USA) was used to generate the figures.

## Results

### Clinical features of the study patients

In total, 108 patients were included in the present study after excluding 8 cases with immunohistochemistry technical failure. For the entire cohort, the median age was 52 years (mean of 51.8 years and range of 26–79 years), and 37.0% of the patients were 50 years or younger. Nine patients had a personal history of cancer and/or synchronous cancer. Of these patients, four were diagnosed synchronously with a malignancy of the endometrium (*n* = 3) or colon (*n* = 1). The patient with synchronous colon and ovarian cancer developed endometrial cancer 2 years later. A previous history of breast cancer and thyroid cancer was noted in two patients and one patient, respectively. The remaining two patients had metachronous urothelial cell cancer and lung cancer after the diagnosis of ovarian CCC. In addition, 23 patients reported a family history of cancer, mostly colorectal cancer (*n* = 4), pancreatic cancer (*n* = 3) and urothelial cell cancer (n = 3).

Table 1 shows that 62.3% of the patients presented with early-stage disease (FIGO I + II), and most of these patients (45.3%) presented with FIGO stage I. Moreover, 25.9% of the patients had concurrent endometriosis, and 91.2% of patients had residual disease ≤1 cm. For the patients with advanced disease, the debulking results indicated that 41.7% of the patients (15/36) had no gross residual disease and that 66.7% of the patients (24/36) had residual disease ≤1 cm. Concerning the chemotherapy response, platinum-sensitive recurrence accounted for 69.6% of the patients.

**Table 1:** Clinical features of the study population (*n* = 108)

Age at diagnosis	52 (26–79)
Personal history of cancer	8.3% (9/108)
Family history of cancer	21.3% (23/108)
FIGO Stage	
FIGO stage I	45.3% (48/106)
FIGO stage II	17.0% (18/106)
FIGO stage III	29.2% (31/106)
FIGO stage IV	8.5% (9/106)
Extent of debulking	
Residual disease = 0 cm (%)	79.4% (81/102)
Residual disease ≤1 cm (%)	88.2% (90/102)
Platinum response	
Platinum-sensitive	69.6% (71/102)
Platinum-resistant	30.4% (31/102)
Follow-up time (mean, range)	46 (1–178)
Disease status at last follow up	
Dead (%)	46.1% (48/104)
Alive with disease (%)	20.2% (21/104)
No evidence of disease	33.7% (35/104)

*Abbreviations*: *FIGO* The International Federation of Gynecology and Obstetrics

### Clinicopathological features of patients with defective MMR

**Fig. 1:**
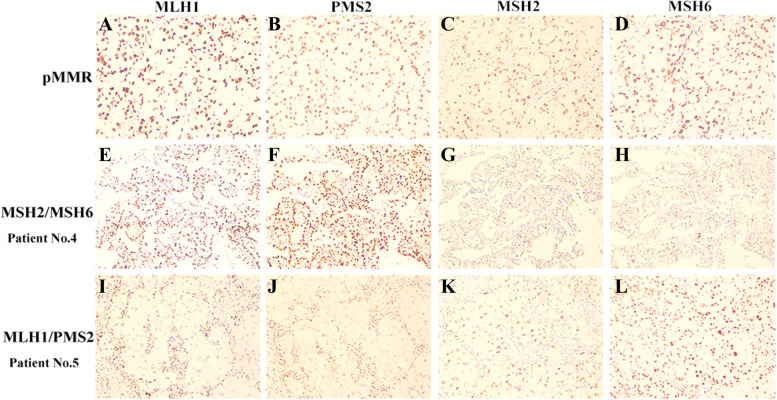
Immunohistochemistry of patients with proficient mismatch repair (1st row, **A**-**D**), indicating loss of MSH2/MSH6 protein expression in patient 4 (2nd row, **E**-**H**) and loss of MLH1/PMS2 protein expression in patient 5 (3rd row, **I**-**L**). Abbreviations: pMMR, proficient mismatch repair

A total of six (5.6%) patients harbored abnormal MMR expression, including MSH2/MSH6 (*n* = 4) and MLH1/PMS2 (*n* = 2) (Fig. 1). Table 2 shows the clinical and pathological characteristics of ovarian CCC patients with deficient MMR. The average age of the six MMR-deficient patients was 45.6 years, which was younger than patients with MMR-intact tumors (average age of 52.1 years), but statistical significance was not achieved (*P* = 0.153). In addition, the rate of MMR-deficient tumors in women ≤50 years was relatively higher than that in those over 50 years (10.0% vs. 2.9%; *P* = 0.266, continuity correction chi-square) (Supplementary Table 1). In terms of personal history of cancer, half of the patients with deficient MMR were diagnosed with synchronous or metachronous cancer, which was significantly higher than their counterparts with intact expression (*P* = 0.002, continuity correction chi-square) (Supplementary Table 1). Two patients had synchronous endometrioid endometrial cancer (No. 3 and No. 5). One patient (No. 2) had an accidental diagnosis of ovarian CCC during the scheduled colorectal cancer surgery and was diagnosed with metachronous endometrioid endometrial cancer 2 years later. A family history of cancer was reported in two patients (No. 3 and No. 6). All six patients had early-stage (FIGO I + II) tumors at first diagnosis. Concerning platinum response, the majority (5/6, 83.3%) of the patients were platinum sensitive.

**Table 2:** Clinicopathological features of ovarian clear cell carcinoma patients with deficient mismatch repair protein

No	Age (years)	Personal history	Family history	Stage	Platinum response	Status	PFS (months)	OS (months)	dMMR pattern
1	51	/	/	IC	Sensitive	NOD	71	71	MSH2/MSH6
2	47	Colon, endometrial	/	IA	Sensitive	NOD	30	30	MSH2/MSH6
3	50	Endometrial	Endometrial, colon, pancreatic	IIB	Sensitive	NOD	30	36	MSH2/MSH6
4	54	/	/	IIB	Sensitive	AWD	19	126	MSH2/MSH6
5	30	Endometrial	/	IIB	Sensitive	NOD	57	57	MLH1/PMS2
6	42	/	Colon	IIA	Resistant	AWD	4	33	MLH1/PMS2

*Abbreviations*: *PFS* Progression-free survival, *OS* Overall survival, *dMMR* Deficient mismatch repair, *AWD* Alive with disease, *NOD* No evidence of disease

The two patients with a family history underwent subsequent genetic testing. Patient No. 6 was demonstrated to carry the following MHL1 germline mutation: c.1756G > C (p. Ala586Pro) (Class 5, pathogenic [23]). Patient No. 3 had synchronous endometrial cancer and ovarian CCC at diagnosis. In addition, patient No. 3 had a significant family history as follows: her mother had endometrial cancer, and two brothers of her mother had colorectal and pancreatic cancer. She was found to have a complex gene rearrangement in MSH2, which has never been reported (Class 3, uncertain significance [23]). Further multiplex ligation-dependent probe amplification tests were negative. Based on the gene mutation test, the diagnosis of underlying Lynch syndrome for patient No. 3 should be made with caution despite the clinical history.

### Survival analysis

**Fig. 2:**
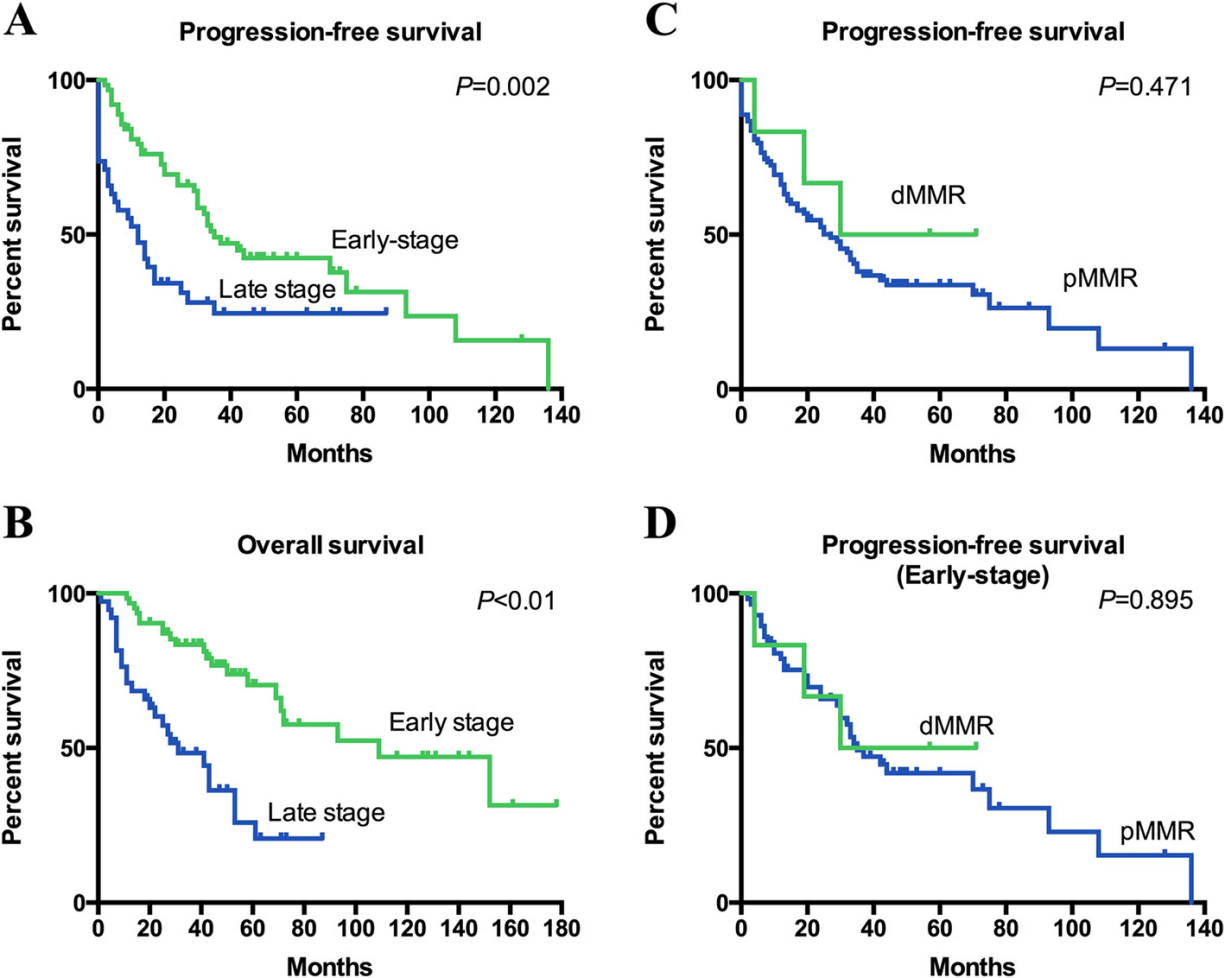
Kaplan–Meier survival curves based on stage and mismatch repair status. Abbreviations: dMMR, deficient mismatch repair; pMMR, proficient mismatch repair

Follow-up information was available in the majority of the patients (96.3%, 104/108). After a mean follow-up time of 46 months (range, 1–178 months), 46.1% of patients (48/104) died of disease, 20.2% of patients (21/104) were still alive with disease, and 33.7% of patients (35/104) had no evidence of disease. Of note, 26 patients had a follow-up time less than 24 months. Of these, six patients were alive without disease, and the remaining 20 patients all died. Figure 2 shows the survival curves for the entire cohort stratified by stage. The median PFS of patients with early and late disease was 35 and 12 months (*P* = 0.002), respectively (Fig. 2A). Similarly, the median OS of patients with early-stage tumors was significantly better than that of patients with advanced disease (109 vs. 31 months, *P* < 0.001) (Fig. 2B). The estimated 5-year overall survival was 70.3% in patients with early-stage tumors and 20.7% in those with advanced disease.

We next evaluated the prognostic implication of MMR deficiency in the study population. The median PFS was higher in patients with abnormal MMR expression than in those with intact expression (54 months vs. 27 months), but statistical significance was not achieved (*P* = 0.471) (Fig. 2C). Considering that all patients with MMR deficiency had early-stage tumors, we further evaluated the prognostic impact of MMR deficiency in patients with early-stage tumors (Fig. 2D), but statistical significance was not achieved in terms of PFS. Overall survival comparison was not made given that all the patients with MMR deficient tumors were still alive (data censored) at last contact.

## Discussion

Several recent publications focusing on MMR evaluation in ovarian CCC are summarized in Table 3. Five of the eight studies included relatively small sample sizes due to disease rarity [13–16, 18]. The prevalence of MMR deficiency ranges from 0 to 13% [11–18]. Bennett et al. conducted the largest series on whole section slides and correlated MMR expression to histological features [11]; they reported that diffuse intratumoral stromal inflammation and the presence of peritumoral lymphocytes may be associated with MMR loss in ovarian CCC [11]. Another study with a large sample size that assessed PROMISE algorithm-related markers, including MMR, in ovarian CCC by tissue microarray [17] has demonstrated a low frequency of abnormal MMR (2%) and no pathogenic DNA polymerase ε (POLE) mutation [17].

**Table 3:** A review of recent studies focusing on MMR immunohistochemistry in ovarian clear cell carcinoma in chronological order

Study	Country	dMMR Rate	Sample	Pattern
Bennett et al.	USA	6% (6/109)	Whole section slides	MSH2/MSH6 (3), MLH1/PMS2 (1), MSH6 (1), PMS2 (1)
Rambau et al.	Canada	2.4% (4/164)	Tissue microarray	MSH2/MSH6 (3), MSH6 (1)
Willis et al.	USA	13% (3/23)	Whole section slides	MSH2/MSH6 (3)
Stewart et al.	Australia	6% (2/32)	Whole section slides	MSH2/MSH6 (2)
Howitt et al.	USA	10% (3/30)	Whole section slides	Not mentioned
Xiao et al.	China	4% (2/50)	Tissue microarray	MLH1/PMS2 (1), PMS2 low (1)
Parra-Herran et al.	Canada	2% (2/90)	Tissue microarray	MSH2/MSH6 (1), MSH6 (1)
Fraune et al.	Germany	0/23	Tissue microarray	/
Our study	China	5.6% (6/108)	Whole section slides	MSH2/MSH6 (4), MLH1/PMS2 (2)

*Abbreviations*: *dMMR* deficient mismatch repair

Universal testing of MMR in ovarian cancer is not routine in most hospitals, and the National Comprehensive Cancer Network (NCCN) guidelines recommend it as clinically indicated. Not surprisingly, higher frequencies of defective MMR have been reported in younger patients in several studies [9, 11, 12, 24]. Rambau et al. tested MMR proteins in 612 ovarian cancer patients by tissue microarray and found that deficient MMR is related to age < 50 years, synchronous endometrial endometrioid cancer and absence of ARID1A [12]. A previous study with a relatively large sample size focusing on ovarian CCC alone (*n* = 109) has shown that patients with abnormal MMR expression are significantly younger with a mean age of 40 years compared to 53.2 years for the overall cohort [11]. In the present cohort, the mean age of the six patients with loss of MMR expression was 45.6 years compared to 52.1 years for patients with MMR-intact tumors, but this difference was not statistically significant. Nevertheless, we noted a rate of 10.0% MMR deficiency in patients aged 50 years and younger.

MMR-deficient tumors have peculiar clinical behaviors, including early-onset metastatic potential, but generally favorable prognosis and remarkable responses to immune therapy [25]. The possible clinical implications of MMR deficiency in ovarian CCC have been evaluated in the literature [11, 12, 17]. However, no consensus has been reached, mainly due to disease rarity and the low frequency of MMR abnormalities. In the present study, six MMR-deficient patients had early-stage disease, which was consistent with the finding that MMR-deficient colorectal cancers are strongly enriched in the early stages of diagnosis [26]. Moreover, patients with loss of MMR expression tended to have longer progression-free survival than those patients with preserved expression, but the difference was not significant. Although the present study was based on a small number of cases, our findings suggested that MMR-deficient tumors may confer a good prognosis in ovarian CCC. Similarly, the prognostic implication of MMR deficiency in ovarian CCC has been evaluated in two studies [11, 17], but no conclusion has been reached. Stewart et al. reported that two patients with advanced tumors harboring MMR abnormalities were alive at 160 months and 124 months following surgery [14].

To the best of our knowledge, the present study represents one of the largest series measuring MMR proteins in ovarian clear cell carcinoma patients. However, the present study had several limitations. First, considering disease rarity, we collected the cases over a long period of time, which led to missing data. Second, the cohort may be limited by the selection and surveillance biases often associated with studies from a single institution. Last, the present study did not evaluate the specific regimen of treatment, which may be a confounding factor for survival outcome.

## Conclusions

The present study showed MMR loss in 5.6% of unselected tumors of ovarian clear cell carcinoma, but this rate increased to 10% when selecting for age (50 years and below). All patients presented with early-stage disease, and half of the patients had synchronous/metachronous endometrial/colorectal cancer. Patients with MMR deficiency seemed to have better progression-free survival when all patients were analyzed, but the difference was not as significance when only the early-stage group was investigated. Thus, additional studies are required to determine whether patients harboring MMR abnormalities have a favorable prognosis.

## Supplementary Information

**Additional file 1: Supplementary Table 1.** Clinical features of the study population (*n* = 108).

## Data Availability

The dataset supporting the conclusions of this article is available upon request. Please contact Prof. Huijuan Yang (huijuanyang@hotmail.com).

## Abbreviations

MMR: Mismatch repair
CCC: Clear cell carcinoma
