# Supplementary material for: The frequency and clinical implication of mismatch repair protein deficiency in Chinese patients with ovarian clear cell carcinoma
Source: BMC Cancer. 2022 Apr 23;22:449. doi: 10.1186/s12885-022-09588-z (PMC9035241; doi:10.1186/s12885-022-09588-z)
Supplement: Supplementary file 1 — Additional file 1: Supplementary Table 1. Clinical features of the study population (n = 108). [file 12885_2022_9588_MOESM1_ESM.docx]

| Supplementary Table 1 Clinical features of the study population (n=108) | | | |
| --- | --- | --- | --- |
|  | dMMR patients  (n=6) | pMMR patients  (n=102) | *P* value |
| Age at diagnosis | | | |
| Age≤50 years | 4 | 36 | 0.266^*^ |
| Age > 50 years | 2 | 66 |  |
| Synchronous or metachronous cancer | | | |
| Yes | 3 | 6 | 0.002^*^ |
| No | 3 | 96 |  |
| FIGO stage | | | |
| Early stage (FIGO I+II) | 6 | 60 | 0.126^*^ |
| Late stage (FIGO III+IV) | 0 | 40 |  |
| Platinum response | | | |
| Platinum-sensitive | 5 | 66 | 0.767^*^ |
| Platinum-resistant | 1 | 30 |  |
| Abbreviations: FIGO: The International Federation of Gynecology and Obstetrics; dMMR=deficient mismatch repair; pMMR= proficient mismatch repair.  ^*^ Continuity correction Chi-square | | | |
